# The Woman’s Medical College of Georgia

**Published:** 1890-06

**Authors:** 


### THE WOMAN'S MEDICAL COLLEGE OF GEORGIA.

This institution begins its second annual term October 1st next, under the Presidency of Dr. A. G. Thomas, of this city. The class will consist exclusively of ladies. Half rates to wives and daughters of physicians, clergymen and Confederate veterans. For information, address J. W. Stone, M. D., box 215, Atlanta, Georgia. 3t

